# Challenges on sexual health communication with secondary school learners, Limpopo province

**DOI:** 10.4102/curationis.v46i1.2321

**Published:** 2023-04-19

**Authors:** Humbulani S. Munyai, Lufuno Makhado, Dorah U. Ramathuba, Rachel T. Lebese

**Affiliations:** 1 Department of Advance Nursing Science, School of Health Sciences, University of Venda, Thohoyandou, South Africa; 2 Department of Public Health, School of Health Sciences, University of Venda, Thohoyandou, South Africa; 3 School of Health Sciences, University of Venda, Thohoyandou, South Africa

**Keywords:** parents, sexuality, sexuality education, learner, parent–child relationship, risky sexual behaviours

## Abstract

**Background:**

A conversation about sexuality is most likely to encourage healthy and positive sexual practices while reducing risky sexual behaviour among adolescents. Traditionally, sexuality is discussed in hushed tones in proverbs and is reserved for adults. On the other hand, adolescents must be well informed about their sexuality to assist them to make informed decisions about their sexual behaviour.

**Objectives:**

The study determined parents’ views regarding challenges of sexual health communication among secondary school learners in the Limpopo province.

**Method:**

A qualitative, exploratory-descriptive and contextual approach was employed for the study. Fifty-six parents were purposively selected, resulting in five focus group discussions that had 8–12 members. One central question was asked, and based on the participants’ responses, probing questions followed. Data were analysed using thematic analysis. Trustworthiness and ethical considerations were ensured.

**Results:**

Three themes, namely communication concerns, role shifting in imparting sexuality education and poor parent–child relationships, and eight subthemes emerged from the data.

**Conclusion:**

This study identified that communication concerns influence parent–child dialogue on sexuality education. Therefore, there is a need to address factors hindering communication such as cultural barriers, role shifting in imparting sexuality education and poor parent–child relationships. This study suggests that parents should be empowered in dealing with children’s sexuality.

**Contribution:**

Parents should be equipped with reproductive knowledge to enable them to talk freely about sexuality with their children. This should be complemented with broader programmes aimed at promoting sexual health education within the traditional family institution.

## Introduction

Traditionally, sexuality is discussed in hushed tones in proverbs and is reserved for adults. On the other hand, adolescents require information about their sexuality to make informed decisions about their sexual behaviour (Baku et al. 2018:1). Science-based, realistic, nonjudgemental information about sex and relationships is provided in sexuality education at an age-appropriate, culturally relevant level (Kemigisha et al. 2018:2; Leung et al. 2019:2). Promoting sexuality education through parent–child communication is a positive and effective strategy for achieving long-term behavioural change and thus a reduction in unintended pregnancy. Sexuality education can reduce sexually transmitted infections (STIs), early sexual debut, multiple sex partners and low, inconsistent condom use (Bonjour & Van der Vlugt 2018:14; Daminabo, Teibowei & Agharandu 2022:65; Pleaner et al. 2022:4). However, parents do not often discuss sexuality with their children because it is embarrassing and uncomfortable to communicate (Rodgers et al. 2018:628).

Globally, parent–child communication is recognised as a crucial strategy to reduce sexual health risks (Othman et al. 2020:313). Sexuality communication plays a vital role in the preparation of teenagers for a safe, productive and fulfilling life. Effective and positive communication between parents and their children about sexual health helps adolescents to establish individual values and make sexually healthy decisions (Venketsamy & Kinear 2020:2). A number of studies have shown that learners who have not been taught sexuality are more likely to engage in high-risk sexual behaviours than those who have been taught sexuality. Learners who received sexuality education were less likely to have several sexual partners, participate in unprotected sex, or become pregnant as teenagers. Additionally, they frequently use condoms or other contraceptive methods (Ram, Andajani & Mohammadnezhad 2020:2).

Despite the evidence that parent–child communication is globally recognised and that parents are acknowledged as the primary source of information that can best influence decision-making responsive to the adolescent’s needs, however, it is still a hurdle because of various sociocultural and religious challenges such as lack of communication skills, low self-efficacy, ignorance and a lack of concrete information and parental underestimation of the child’s sexual behaviour (Aventin et al. 2020:3). Learners are explorative, and with the advent of technology and social media content, they are usually misinformed as they do not know the right information. Döring (2021:9) indicates that learners may need help and support with sexuality information and strategies to build their confidence.

In African countries, culture turns out to be a barrier for parents to openly discuss sexuality with their children. Modise (2019:85) indicated that parents felt uncomfortable and believed that discussing sexuality was taboo. However, some parents discussed sexual and reproductive health with children, especially female parents. This is an indication that women are closer to their children as primary caregivers and men are culturally detached from their parental role, as they are only seen as providers. Research suggests that children’s age and gender do predict interaction of sexual communication with parents; parents are more likely to share messages with female than male teens, focusing on abstinence and resisting a partner’s advances, while other research shows that teen girls are more likely than boys to talk with family members about sex (Grossman, Jenkins & Ritcher 2018:6). Parents who browsed the subjects preferred to address a few topics such as abstinence, menstruation and human immunodeficiency virus (HIV) and acquired immunodeficiency syndrome (AIDS). Dioubaté et al. (2021:5–6) highlighted that condom use and contraception were hardly discussed because parents think that talking about such issues is promoting sexual immorality or encouraging children to engage in sexual relationships (Shams et al. 2017:5).

### Problem statement and research objectives

Sexuality education is regarded as a cultural taboo subject by most African people, especially among black communities. Society feels that it is not appropriate to open such conversations with children; however, learners still get information from friends and social media. Literature has indicated that the timing of education, the lack of knowledge of parents and their reluctance in communicating with learners has resulted in sexual ill-health and misbehaviours of sexual activities. The South African government saw the importance of sexuality education and introduced life orientation in schools, because parents were reluctant to communicate with learners and shifted the responsibility to the schools. However, the status quo remains because teachers are parents from the same communities affected by sociocultural and religious barriers and fail to provide comprehensive sexual education. The involvement of the parents might have the potential to impact adolescents in decision-making and is often referred to as a primary and preferred source of sexual health information. Parents can play an important role in supervising learners’ activities as primary caregivers by conveying appropriate sexual health information in a respectful manner. In relation to sexual health practices, role models can exert a considerable influence on adolescents’ attitudes, values and beliefs.

For that reason, the researchers aimed to determine parents’ views regarding challenges of sexual health communication among secondary school learners in the Limpopo province. The objectives of the study were to explore and describe parents’ views regarding challenges of sexual health communication among secondary school learners in the Limpopo province.

## Research design and method

This study employed a qualitative, explorative, descriptive and contextual design. The study aimed to explore and describe parents’ views regarding challenges of sexual health communication among secondary school learners in the Limpopo province. The study design was chosen as it provides an extensive discovery of information about promoting sexuality education for Grade 8 learners and was found suitable for this study.

### Setting

Mopani and Vhembe Districts in the Limpopo province were the study areas. Mopani District is located on the eastern side of the Limpopo province, bordering Mozambique, while Vhembe District is located on the northern side of the Limpopo province, bordering Zimbabwe (municipalities.co.za n.d.). These two districts were purposefully selected because of the high sexual health issues that are prevalent in the district that might be attested to poor sexual health communication. The researcher approached public secondary schools with the aim of accessing a population of parents through the school governing bodies and committees.

### Population and sampling (participants)

The target population were all parents with learners in the school where the study was conducted. Nonprobability purposive sampling was employed to recruit parents who were willing to be part of the study with the assistance of the principals and school governing bodies. The sample consisted of 56 participants from the eight schools within the two districts, resulting in a total of five focus groups.

### Data collection

Data were collected from selected schools after making appointments with participants. Each meeting lasted for 1 h – 1.5 h. A conducive climate was created for everyone to feel free to share their challenges, and the seating was arranged in a semicircle. Each focus group had 8–12 members. Data were collected by means of a focus group discussion, using an unstructured interview method. The purpose of using this method was to understand parents’ views regarding challenges of sexual health communication among secondary school learners in the Limpopo province. Different methods to enhance trustworthiness of data were used such as taking notes, observation and using an audiotape to reduce bias (De Vos et al. 2017:361). The focus group started with a central question: ‘can you share your challenges regarding sexual health communication with your children?’ The central question was asked to all five focus group discussion groups, followed by probing questions because the researcher wanted to understand more about their challenges regarding sexual health communication. Data saturation was achieved when no new information was upcoming from the focus group discussions, and there was no substantial addition to the codes and themes being developed (Brink, Van de Walt & Rensburg 2017:193). Termination of the focus group interviews occurred after a maximum of two visits for some three visits. Data were collected for a period of three months (May 2019 – July 2019).

### Data analysis

The data were analysed conceptually, which included reading, coding and developing themes (Brink et al. 2017:193). Raw data were transcribed verbatim, including observational notes collected from the focus group discussions. The data were condensed and organised into themes and subthemes to make sense. The researcher approached an experienced qualitative data coder to analyse the data again, then an agreement was achieved. Literature control was presented after data were analysed to compare findings of this study (Brink et al. 2017:193).

### Measures to ensure trustworthiness

The present study used the model of Lincoln and Guba (1985) to ensure trustworthiness. This model is characterised by four strategies for ensuring trustworthiness: credibility, transferability, dependability and confirmability. Credibility was ensured through prolonged engagement with the participants, as more than one visit was made for focus group discussion interviews, and during transcription of data, when clarity was needed, the researcher contacted the participants for further clarity; furthermore, field notes and observational notes were captured to enhance credibility. Transferability is the extent to which the results can be transferred to other contexts or settings; this was ensured by the purposive sampling technique to make sure that the selected participants were representative of the different views of parents across the different settings in the Limpopo province. To enhance the dependability of the data, an audit trial was used where a track record of the data collection process was developed, and during analysis, field notes and observations written during data collection were compared with data and corroborated with the findings of this study (Brink et al. 2017:172). Confirmability is the extent of the confidence that the results would be confirmed or corroborated by other researchers; this was achieved through reflexivity, where weekly meetings with promoters and independent coder were held after data analysis to reflect on the transcripts and themes. Feedback from the promoters and independent coder confirmed that all the quotes used in the participants’ transcripts supported the identified themes.

### Ethical considerations

#### Permissions

Ethical clearance was granted by the University of Venda Human and Clinical Trial Research Ethics Committee (HCTREC) (reference number SHS/19/PDC/37/2410). Provincial Department of Education Limpopo province and the district managers and principals granted permission. It was agreed that the researcher would not visit schools during examination time and would not disrupt classes. A written informed consent form was obtained from each participant.

#### Consent

Consent is morally justified primarily on the basis of autonomy, as research participants’ autonomy can be protected and supported through the consent process. A brief explanation of the research purpose and the fact that participants are not forced to participate were given to the participants. Based on the information given to them, they made a free choice.

#### Confidentiality and anonymity

Once the researcher has information, confidentiality pertains to what the researcher does with it, specifically how much he or she discloses to others. The researcher gave an assurance that data would be reported anonymously; anonymity, in contrast, is concerned with the attribution of information. Participants were informed that the researchers would maintain their anonymity and would not report actual names or other identifying information. There is no control over participants breaking internal confidentiality in focus groups, but the researcher relies on the following ground rules and adheres to consent procedures. As the researcher explains the purpose of the study, he or she also explains that no information should be discussed outside the focus group meeting.

## Results

The following themes emerged during data collection: communication concerns, role shifting in imparting sexuality education and poor parent–child relationships. Eight subthemes also emerged. The narratives of parents’ views are presented as direct quotations of participants. Themes and subthemes are indicated in Table 1.

**TABLE 1:** Themes and subthemes that emerged from data analysis.

Themes	Subthemes
Communication concerns	Task shifting of responsibility
	Cultural barriers
	Uncertainty of time or age at which to impart knowledge
	Fear of embarrassment
	Reactive sharing of information instead of being proactive
Role shifting in imparting sexuality education	Parents shifting responsibility to school and religious institutions
Poor parent–child relationships	Parents lack confidence in the subject
	Reluctance and avoidance of sexual health discussion

### Demographic characteristics of participants

The study included 56 participants in focus groups consisting of parents from the Greater Giyani and Thulamela municipalities. Parents were aged between 35 and 63 years, both female and male. Most of the participants were female (*n* = 42) and a minority were male (*n* = 14). The majority of participants were not employed. The highest qualification held by three participants was a degree; 24 participants did not have a Grade 12 certificate.

Themes and subthemes identified in the study are discussed in the following sections, supported with direct quotations and the literature.

#### Theme 1: Communication concerns

Participants expressed that communication concerns inhibited parent–child dialogue about sexuality. However, some participants considered the provision of sexuality education only under conditions, while others did not. Communication concerns about promoting sexuality education were further classified into five subthemes, namely: task-shifting of responsibilities, cultural barriers, the uncertainty of time or age to impart knowledge, fear of embarrassment, increased temptation and communication after things go wrong.

**Subtheme 1.1: Task shifting of responsibilities:** In this study, participants indicated that parent–child communication is lacking because they shift their responsibility to teach learners about sexuality. A parent assigns a family member such as a spouse, siblings or elders in the family to discuss information about sexuality with their children. One participant said:

‘My wife is the one who is supposed to talk with children and only report to me [*husband*] back the child who is naughty.’ (FGD 4 – P7, female, 47 years old)

Another participant concurred:

‘I would rather have another person talk to my child on my behalf. I believe that the other person will be much more open than me because I am afraid the child will disrespect me.’ (FGD 1 – P3, male, 44 years old)

Task shifting has been observed in communities where parents as primary caregivers are no longer staying with their children and have shifted the responsibility to the grandparents, especially grandmothers.

**Subtheme 1.2: Cultural barrier:** Participants expressed that cultural barriers are a challenge that restrict parents from communicating with their learners. Participants indicated that it is taboo and insulting for adults to discuss sexuality matters with persons younger than them.

One participant said:

‘Culturally, we are not allowed to talk about sex-related issues with young people.’ (FGD 3 – P4, female, 38 years old)

Another participant said:

‘In Venda culture, for us to talk to your child about sex, especially my daughter, is a taboo.’ (FGD 4 – P2, female, 53 years old)

Traditionally, in many African cultures, sexual health communication is considered highly inappropriate when parents talk about sexuality matters but emphasise abstinence without explanations.

**Subtheme 1.3: Uncertainty of time or age at which to impart knowledge:** This study revealed that uncertainty of time or age to impart knowledge about sexuality leaves some participants unsure about the age to have such a discussion with their children. Participants indicated that it is challenging to impart sexuality matters to Grade 8 learners because they believe that learners are still young. The information may influence learners to become sexually active. A participant said:

‘I do not know when the right time is and how to start teaching my child.’ (FGD 6 – P2, female, 54 years old)

Another one said:

‘I cannot talk about sex with my children. They are still young. I am unsure if I can start now.’ (FGD 3 – P2, female, 37 years old)

Appropriate age-specific time is difficult to determine, as parents underestimate the sexual activity of their children; recent observations reveal early sexual debuts and incidences of teen pregnancies among the ages of 10–12 years.

**Subtheme 1.4: Fear of embarrassment:** Parent–child communication is limited by fear of embarrassment and parents’ lack of knowledge about sexual matters. Participants pointed out that it is embarrassing to answer sexually intimate questions. When is the right time to have an intimate relationship? One participant indicated that:

‘I did not know what to say when my daughter asked me when the right time was to start dating. I felt embarrassed to answer her because it is not culturally accepted. Although I know I should tell her the truth.’ (FGD 5 – P2, female, 56 years old)

Another participant said:

‘Talking about contraception is embarrassing. As a parent, it was difficult for me to explain.’ (FGD 4 – P3, female, 43 years old)

Parents highlighted that sexuality communication is a shameful and embarrassing topic because it is usually associated with humiliation, self-guilt and stigma by society.

**Subtheme 1.5: Reactive sharing of information instead of being proactive:** Reactive sharing of information is viewed as an obstacle to promoting sexuality education. The study results revealed that parents are hesitant and feel unprepared for and uncomfortable communicating about sexuality with their children. Topics such as intimate relationships, pregnancy and contraception are discussed after parents realise that their children are sexually active or pregnant. An individual participant supported by the group had this to say:

‘The challenge is that we start to talk to a girl child when realising that the child is pregnant. Then the whole family gathers to talk about and with the pregnant child. We wait until the child has become pregnant. We only talk when it is no longer useful.’ (FGD 2 – P1, female, 56 years old)

One participant added that:

‘At our school, a certain pastor was invited to talk with learners after the school management realised that 20 learners were pregnant.’ (FGD 3 – P1, male, 58 years old)

Another participant said:

‘I have communicated with my daughter while she was in grade 6 to see that she is in the puberty stage. We talked about menstruation, how to take care of menstruations, abstinence to prevent pregnancy.’ (FGD 4 – P2, female, 58 years old)

Providing genuine and respectful communication in a genuine manner is very important because learners will be able to receive such information in a good way without feeling judged. However, parents usually comment on issues in a passive manner and when a sexual problem has occurred.

#### Theme 2: Role shifting in imparting sexuality education

Participants found it particularly difficult to discuss sexually related matters. However, participants acknowledged their role in imparting knowledge about sex education but shifted that role to teachers and other professionals such as nurses and priests. The following subtheme emerged: Parents shifting responsibility to school and religious institutions.

**Subtheme 2.1: Parents shifting responsibility to school an d religious institutions:** Shifting roles to schools and churches was cited as a challenge regarding parent–child communication. Some participants believed that the provision of information about sexuality is the role of the teachers. Most participants highlighted a lack of knowledge and skill as a problem that shifted their primary teaching function to teachers. Participants felt there was no reason for them to talk about sexuality at home because learners are taught at school in the subjects of life orientation and life science. An individual participant said:

‘I thought it is the role of the teachers to teach … so they are going to teach children according to the curriculum and age of the learner unlike at home.’ (FGD 5 – P2, female, 35 years old)

Another participant said:

‘Teachers must continue teaching because parents do not have sufficient information about sex education and different types of contraceptives. I do not have that information.’ (FGD 1 – P3, male, 42 years old)

Some participants indicated that it is difficult to have dialogue with their children because their parents did not discuss this topic with them as they grew up. An individual participant supported by the group said:

‘During my school time, my parents did not tell us anything about sexuality. I was taught about menstruation in school when studying biology. So, I expect teachers to teach them likewise.’ (FGD 4 – P4, female, 52 years old)

‘I do not bother myself about teaching my children about sexuality because in our church they conduct workshops to guide youth on how to conduct themselves regarding their sex life and relationships.’ (FGD 3 – P5, female, 38 years old)

Another participant supported by saying:

‘Children who are being guided at church have good morals. Pastors must emphasise sexuality.’ (FGD 5 – P2, female, 43 years old)

When parents fail to take their primary role of teaching, they shift their role to schools and churches, while these institutions and parents need to work together to communicate sexual health information to learners. However, Christian parents turn to relax, thinking that the church will teach their children about sexuality. Albeit the church only emphasise abstinence to reduce sexual risks, which has failed the learners.

#### Theme 3: Poor parent–child relationships:

Parents play a major role in the lives of their children; they should model healthy sexual practices. Literature asserts that children who relates well with their parents usually make good sexual health decisions.

Two subthemes emerged: parents’ lack of confidence in the subject and reluctance and avoidance techniques of the subject.

**Subtheme 3.1: Parents’ lack of confidence in the subject:** Participants indicated a lack of confidence in communicating sexual health information because of poor parent–child relationships. Culturally, mothers are closer to their daughters than their sons, which makes it difficult to broach such a subject.

An individual participant said:

‘It is difficult to discuss sexuality. Maybe it is because of my relationship with my son.’ (FGD 4 – P6, female, 42 years old)

Another participant said:

‘I am not confident to confront my children about sexuality education. It is difficult for me to talk or teach my son, but one person whom he relates well with is his elder brother.’ (FGD 2 – P7, male, 37 years old)

Developing good parent–child relationships contributes to a positive, open sharing of information; respecting the learner as an individual encourages the learner to develop better sexual health values.

**Subtheme 3.2: Reluctance and avoidance of sexual health discussion:** Participants verbalised that they are reluctant and avoid communicating about sexuality issues. Participants highlighted feeling embarrassed to watch television with children when people are kissing. Some said they (parents) changed the channel on the social broadcasts:

‘In case of age-restricted shows, we just leave them watching television without supervision to avoid them asking questions.’ (FGD 4 – P2, female, 55 years old)

Another participant added:

‘I avoid making any comment on radio talks or television shows. If we are watching television and something occurs like kissing or sex appears on screen, we look down or try to reach out for a remote to change the channel because we feel embarrassed.’ (FGD 5 – P2, male, 40 years old)

Avoidance and being reluctant is refusing to take responsibility for parenting, because parents need to control and supervise what the children are watching in the media and Internet platforms. Social media and Internet can negatively influence learners to adopt unhealthy sexual choices when parents do not interrogate media issues with them.

## Discussion

This study revealed the views of parents regarding promoting sexuality education for Grade 8 learners in the Limpopo province. They are often embarrassed or feel shy to discuss sensitive topics with their children; hence, they shift the responsibility of communicating with their children about sexuality. However, the burden is shifted to the significant other or other family members. This study further highlighted gender differences in alleviating difficulties in discussing sexuality education. Evans et al. (2019:182) highlighted that mothers discuss sexuality with the girl child and fathers with the boy child. This implies a need to empower parents in communication skills to avoid shifting responsibility. Children who receive information about sexuality from a parent are more likely to be free to discuss sexual matters than learners who never received information from their parents. Those adolescents who are able to communicate with their parents easily are more likely to engage their parents in sexual conversation than adolescents who have trouble talking to them. Klu et al. (2022:7) found similar results. The findings of this study cited cultural barriers as a critical obstacle to parent–child communication about sexuality (Yohannes & Tsegaye 2015:4). Talking about sexuality is taboo. The barrier for parents is perceived as a social taboo on sexuality discussion and a lack of knowledge about the topic (Mbachu et al. 2020:8; Ram et al. 2020:5). This implies that cultural taboos and cultural beliefs about sexuality are deeply embedded in parents’ lives and obstruct communication. Parents often do not openly discuss the subject because it is culturally sensitive, and they lack communication skills, which makes them not feel free to discuss it with their children. This finding was similar to Bikila et al.’s (2021:4) findings that also exposed that parents were not allowed to discuss sexuality. In contrast, Shumlich and Fisher (2020:1118) suggested that clear and unambiguous talks can help reduce sexual risk behaviours and promote healthy adolescent sexual development. The findings further highlighted task shifting to the schools and churches. The study by Mavhandu-Mudzusi and Mhongo (2021:11) revealed that some parents believed that external entities, including the educational system, should bear accountability.

The findings of this study highlighted that the appropriate age for initiating the discussion was cited as the greatest common barrier, as parents were always not aware that their children were sexually active. Communication should relatively respond to participants’ age, and most parents only discuss physiological body changes with children (Johnson 2020:8) and reserve important information such as being intimate, the consequences and responsibility thereof. Parents felt uncomfortable initiating discussions with their children about sexuality because of age uncertainty; findings from various studies revealed that some parents began talking to the learner children as early as 10 years old because their bodies are undergoing physical changes at this age This dialogue is only initiated to protect children from sexual health risks (Thin Zaw et al. 2021:85).

Fear of embarrassment contributed to the lack of parent–child communication; parents felt they were not confident enough to talk about the subjects as they lacked accurate, comprehensive sexual information. It is a common practice in black communities for parents to just say ‘do not play with boys’ without providing accurate and straightforward information (Zulu et al. 2022:23). Parents struggle with their own lack of sexual knowledge and are frequently too embarrassed to discuss sexuality with their children because it is culturally inappropriate, as children are usually sent to the aunt or elder member in the family to talk to the learner child. In support of the current findings, Othman et al. (2020:318), Mullis et al. (2021:399) and Mekonen et al. (2018:5) revealed that a lack of communication is linked to fear, embarrassment at discussing with children and taboos attached to sexuality.

This study highlighted that parents are reactive instead of being proactive; usually parents start talking about issues when something crops up – for example, on seeing a pregnant teenager, they will comment in a negative way for the child learner to realise that it is not acceptable, without directly communicating fruitful sexual education information. This finding is consistent with the findings of Mbachu et al. (2020:7) and Flores et al. (2019:541), which showed that unpleasant events were used as opportunities for parents to talk with their children. Jones et al. (2019:766) also reiterated that parents began commenting about sexuality issues on various occasions about an indecent television scene. However, other researchers articulated that the discussion was limited to reprimands for abstinence (Mabunda & Madiba 2017:170), preserving virginity and avoiding pregnancy Rouhparvar, Javadnoori and Shahali (2022:8.). Abstinence is promoted but it is not realistic, as society has transformed, acculturation has occurred and values and beliefs have shifted. The advent of social media and technology has changed the landscape of sexuality information. This does not benefit the children; rather, it exposes them to sexual health risks. Therefore, it is essential for parents to become proactive regarding the provision of sexuality education.

This study revealed that parents are lacking the knowledge and skills to approach this socially taboo subject. Sexuality education is a comprehensive subject that do not only encompasses HIV, AIDS and STI’s only but a broad range of factors such as responsible dating, negotiating safe sex and choosing the right contraceptives, to mention a few. Therefore, parents need to be empowered to have this information so that they can assist the learner children. This is similar to the findings reported by Ezwenwaka et al. (2020:11) which indicate that parents’ role as educators is hampered by a lack of knowledge and approach on engaging children on sexuality issues (Dagnachew Adam et al. 2020:4–5; Ezenwaka et al. 2020:11).

The findings of this study specified that parents did not feel confident or comfortable in communicating with their children on sexuality, partially because of poor parent–child relationships. In support of this finding, Szkody et al. (2018:2643) argued that an excellent parent–child relationship must be established with the child when they are young, as this will encourage parent–adolescent communication when children are older. The results of the study conducted by Benharrousse (2020:34) suggested that parents were more conservative in giving sexuality education. Maintaining a good parent–child relationship forms a strong foundation of communication skills. This implies that self-confidence builds an individual’s self-esteem. Hence, one could be able to share information. Being shy makes parents ignorant and reluctant to open up about sexuality education. This also applies to parents who have a good relationship with children. They do not encounter the problem of communication with their children about sexuality (Klein, Becker and Štulhofer (2018:1493). Therefore, parents and children need to maintain an excellent relationship to promote therapeutic talks.

### Strengths and limitations

As for strengths of this study, the community liked the lecturer (nurse educator) who collected data, which made parent discussions on such sensitive topics easier. Data analysis was performed in a reiterative process with supervisors to ensure that the yielded themes were fully uprooted from the collected and transcribed data, which assisted in ensuring the findings’ reliability and validity. Apart from these advantages, there were some drawbacks. Although we anticipated that using focus groups rather than in-depth interviews would allow active engagement of participants for more in-depth dialogue and deliberation, those who held minority viewpoints may have felt uncomfortable to freely express themselves or speak up. Furthermore, participants shared theoretical stories than talking directly about their own children to avoid dishonouring their children. Male participants were also more problematic for researchers to probe, so they represent a significant population for future research. It was thought that the in-depth interviews could have been more effective at this early stage for one to gain a more comprehensive perspective on certain issues such as contraceptives, sex education, termination of pregnancy and preferences for when to begin sexuality education. This could be a research topic in the future. Our goal-directed selection method was not intended for generalisability to the Vhembe or Mopani Districts. Rather than reflecting the distribution of both nationalities within the population, the researchers fixated our goal on warranting sufficient sample size to attain acceptable depth of information from participants’ responses and to arrive at the point of data saturation when choosing the sample size.

### Recommendations

This study recommends that the community start forums where sexual health issues are discussed, and parents should be empowered on issues of sexual health. The traditional leadership should revise and revisit the traditional institutions which young boys and girls attend for initiation to include issues of sexual health education. Parents should also be empowered with comprehensive sexual education and services available that support teenagers and adolescents. Parents need to be involved in their children’s lives by engaging in sexual health topics while watching television with them.

The education system should continuously update the curriculum on sexual health education and communicate comprehensive sexual education, unlike limiting it to HIV and AIDS. Health services should also promote the provision of comprehensive sexual health information.

There is a need to address sociocultural taboos and religious beliefs that hinder communication. In addition, strategies must be developed to support parents to have confidence in promoting sexuality education.

## Conclusion

This study disclosed that parent–child conversation towards sexuality health matters was limited if not absent in Mopani and Vhembe Districts. Challenges identified as obstructing sexual health communication were issues such as cultural and religious barriers, uncertainty in imparting knowledge, role shifting in imparting sexuality education and poor parent–child relationships. Open, supportive communication between parents and young people related to sexual and reproductive health matters has the potential to postpone engagement with sexual activity, protect youth from risky sexual behaviours and support the healthy sexual socialisation among youth. This study therefore recommends that effective parent–learner conversations regarding issues around sexuality, the cultural norms and religious beliefs that hinder communication as well as the lack of knowledge and confidence of parents should be addressed. Furthermore, programmes aimed at supporting parents in getting more involved in their adolescents’ lives and to engage in healthier talk with their children about their sexuality need to be applied in the local communities. Educational pamphlets can be of good assistance.
